# Chromatin loops, gene positioning, and gene expression

**DOI:** 10.3389/fgene.2012.00217

**Published:** 2012-10-17

**Authors:** Sjoerd Holwerda, Wouter de Laat

**Affiliations:** Hubrecht Institute, Royal Netherlands Academy of Arts and Sciences, University Medical Center UtrechtUtrecht, Netherlands

**Keywords:** chromatin domains, gene expression, nuclear organization, genome structure, nuclear periphery

## Abstract

Technological developments and intense research over the last years have led to a better understanding of the 3D structure of the genome and its influence on genome function inside the cell nucleus. We will summarize topological studies performed on four model gene loci: the α- and β-globin gene loci, the antigen receptor loci, the imprinted H19–Igf2 locus and the Hox gene clusters. Collectively, these studies show that regulatory DNA sequences physically contact genes to control their transcription. Proteins set up the 3D configuration of the genome and we will discuss the roles of the key structural organizers CTCF and cohesin, the nuclear lamina and the transcription machinery. Finally, genes adopt non-random positions in the nuclear interior. We will review studies on gene positioning and propose that cell-specific genome conformations can juxtapose a regulatory sequence on one chromosome to a responsive gene on another chromosome to cause altered gene expression in subpopulations of cells.

## INTRODUCTION

Only a few percent of the 3.2 billion base pairs of our genome is coding sequence. The remainder is intronic and intergenic sequences, long considered to be junk DNA, but now realized to contain hundreds of thousands of sequence modules with the potential to regulate gene expression (Shen et al., 2012). This greatly outnumbers the ~25,000 genes that we carry in our genome. For the great majority of regulatory sites we do not know though whether they really exert a function *in vivo* and, if so, to which target gene they direct their activity. Studies into the shape of our genome provided evidence that regulatory DNA sequences can control transcription over distance by physically contacting target genes via chromatin looping. Initially such work was primarily done on individual gene loci. We will highlight findings on some of the most studied model gene systems, including the α- and β-globin gene loci, the immunoglobulin and other antigen receptor gene loci, the imprinted H19–Igf2 locus and the Hox gene clusters. Collectively, these studies showed how local DNA topology can change dynamically in time and place to accommodate developmental gene expression. It also uncovered some of the trans-acting factors that fold the chromatin. We will discuss the role of the nuclear lamina, CTCF, cohesin, and RNA polymerase II (RNAPII), being currently the most intensively studied general organizers of chromosome topology. Collectively, all studies emphasize the relationship between genome structure and genome function. Consensus seems to have reached now for shape being crucial for function within the ~1 Mb scale. Here, regulatory sequences need to physically get in contact with genes to control their transcription. Beyond this level of organization, it is not as obvious how relevant the nuclear position and/or genomic environment of genes will be. Studies manipulating the nuclear location of genes start to provide insight in this and will be discussed. Finally, we propose that the probabilistic nature of nuclear positioning implies that we need to move from cell population-based to single cell studies to understand how remote genomic sequences can influence each other’s function.

## FUNCTIONALLY RELEVANT DNA INTERACTIONS BETWEEN GENES AND REGULATORY SEQUENCES

The realization that sequence information required for proper gene expression may sometimes reside at a large chromosomal distance away from the gene body came from observations in patients, showing that the deletion of sequences away from the β-globin genes proper caused thalassemia (Kleinjan and van Heyningen, 2005). For a long time, the mechanisms behind long-range gene activation remained enigmatic. Although still not entirely understood it is now clear that it involves physical contacts between such remote regulatory sequences and the genes that they control. This discovery relied mostly on the development of chromosome conformation capture (3C) technology, a method invented 10 years ago (Dekker et al., 2002) that allows quantitative measurements of DNA contact frequencies between pairs of selected genomic sites. Here, we will highlight observations made by 3C technology on four gene clusters (the globin gene loci, the antigen receptor loci, the imprinted H19–Igf2 locus and the Hox gene loci) that serve as model systems for varying types of gene regulation.

### THE α- AND β-GLOBIN LOCI

**FIGURE 1:**
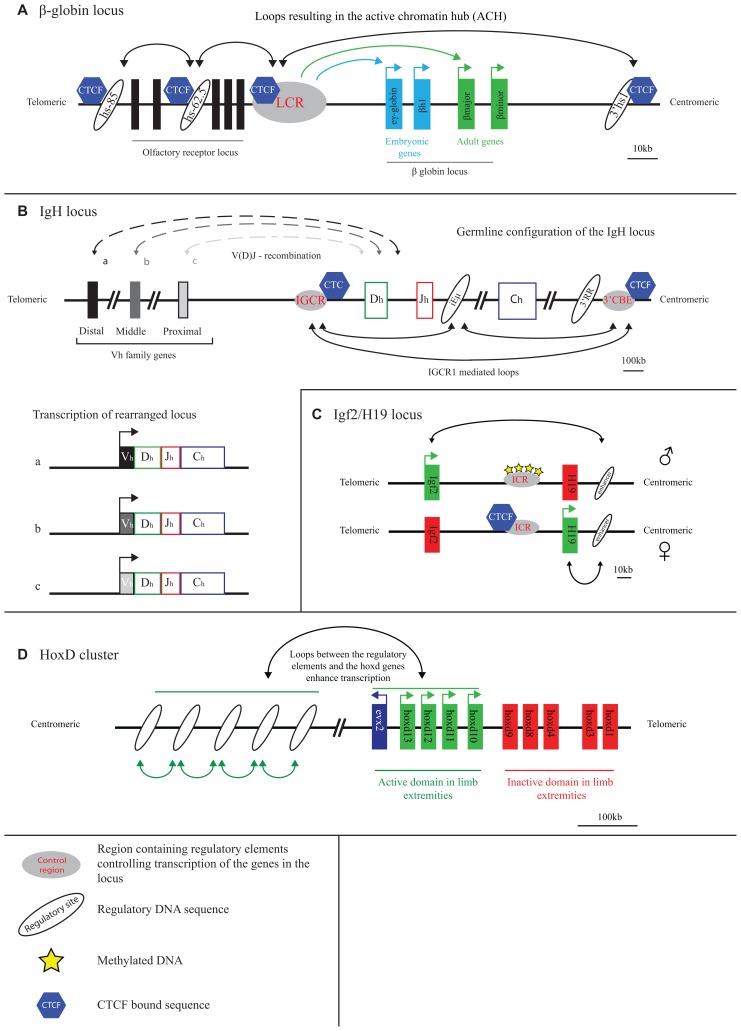
**Long-range transcriptional regulation at model gene loci.** **(A)** At the active β-globin locus, LCR–gene contacts and interactions between flanking CTCF sites set up an active chromatin hub (ACH). **(B)** The IGCR1 contacts the 3′ regulatory region and the intronic enhancer of the IgH locus in pro-B cells. Inclusion of the distal V genes is influenced by the presence of the IGCR1. **(C)** CTCF blocks the interaction of the Igf2/H19 enhancer with the Igf2 gene on the maternal allele. Methylation of the ICR prevents CTCF binding and enables Igf2 expression from the paternal allele. **(D)** A “regulatory archipelago” controls the expression of the hoxd13–hoxd10 genes over distance in limb extremities.

Early evidence for chromatin looping being involved in mammalian gene regulation comes from studies on the β-globin locus. This is perhaps unsurprising as the globin loci have always been the subject of intense gene expression studies: their misregulation underlies thalassemia and the α- and β-globin genes serve as model systems to study developmental gene regulation. As pointed out, the observation that the deletion of sequences away from, but not affecting, the genes proper caused thalassemia (Van der Ploegh et al., 1980) first suggested that gene transcription was controlled by remote regulatory sequences. A series of remote regulatory sites were then demonstrated to exist in these loci, the most important ones in the β-globin locus collectively referred to as a locus control region (LCR). The LCR controls expression of multiple β-globin genes which are arranged on the chromosome in order of their timed expression during development: embryonic β-globin genes are closest to and adult genes are furthest away from the LCR (**Figure 1A**). Proximity on the linear DNA template therefore clearly matters, but the exact mode of LCR action over distance long remained elusive. 3D proximity was implicated in transcription regulation when it was found that linear proximity is no longer important when two genes are positioned together at a large distance from the LCR (Hanscombe et al., 1991; Dillon et al., 1997). In 2002, first direct evidence for chromatin looping and spatial contacts between the LCR and an active β-globin gene was obtained, in studies using RNA TRAP (Carter et al., 2002) and 3C technology (Tolhuis et al., 2002). 3C technology in particular appeared extremely useful for further investigations on the topology of the β-globin locus.

The 3D configuration of the β-globin locus was found to dynamically follow the changes in gene expression that occur during development and during red blood cell differentiation. LCR–gene contacts are not detectable in tissue where the globins are inactive. During development, the LCR switches its contacts from embryonic to adult β-globin genes to ensure their activation at the appropriate developmental stage (Palstra et al., 2003). Proteins were shown to set up the chromatin loops in the locus. Transcription factors such as EKLF, GATA1, and Ldb1, that are important for proper globin gene expression and that bind to both the LCR and gene promoter regions, all appear necessary for stable LCR–gene interactions (Drissen et al., 2004; Vakoc et al., 2005; Song et al., 2007). Another transcription factor, CTCF, forms chromatin loops between binding sites surrounding the locus (**Figure 1A**). These CTCF-mediated loops precede LCR–gene contacts during red blood cell maturation (Palstra et al., 2003). The spatial entity formed in red blood cells as a consequence of LCR–gene and CTCF-mediated DNA interactions was referred to as an active chromatin hub (Tolhuis et al., 2002).

An outstanding question is whether gene activity follows locus conformation or vice versa. The inhibition of transcription was found to not change the chromatin loops, suggesting that function follows structure in the β-globin locus (Mitchell and Fraser, 2008; Palstra et al., 2008). More direct evidence that transcriptional enhancement is a consequence of looping has recently been provided. Ldb1 requires GATA1 for recruitment to the β-globin promoter, but binds to the LCR in a GATA1 independent manner. In an elegant assay employing artificial zinc fingers (ZFs) in GATA1-null cells, the tethering of ZF-Ldb1 to the β-globin promoter was shown to induce LCR–gene contacts and chromatin looping, and to activate β-globin gene expression. Without the LCR, loops were absent and gene expression was not activated (Deng et al., 2012). This data supports the idea that looping toward target genes is crucial for distal enhancers to activate transcription. Interestingly, a truncated version of Ldb1 composed of only its self-association domain was already sufficient to induce chromatin looping and activate transcription initiation, suggesting that Ldb1 multimerization may stabilize contacts between remote globin DNA sequences.

Similar to the β-globin locus, the mammalian α-globin genes are controlled by distal enhancer elements (Sharpe et al., 1993; Gourdon et al., 1994; Higgs et al., 1998). Active histone marks and erythroid-specific transcription factors are present at the locus before the occupancy by RNAPII is measurable (Anguita et al., 2004), suggesting that there is a role for these factors in recruitment of RNA polymerases to the α-globin gene promoters. Looping of the key enhancer elements to the α-globin promoters, with intervening DNA sequences looping out, has been demonstrated (Vernimmen et al., 2007, 2009). Timing of looping coincides with the binding of the pre-initiation complex and elongation factors (Vernimmen et al., 2007). Protein factors like GATA1, Ldb1, and Sp/XKLF also bind to the α-globin genes and regulatory sequences, and can be expected to perform similar roles in chromatin looping and transcription regulation as seen for β-globin.

### ANTIGEN RECEPTOR GENE LOCI

The immunoglobulin loci, which are active in B cells, and the T cell receptor (TCR) loci that are active in T cells, generally stretch over large chromosomal regions of up to 3 Mb and are subdivided into different regions (V, D, J, and C) that each contain multiple gene segments. Particularly the V region is often extremely large. DNA rearrangement via V(D)J recombination is required to combine the different gene segments and assemble a functional antigen receptor that is unique in every B or T cell (Jung and Alt, 2004). The RAG proteins carry out V(D)J recombination and need to physically hold together two target sequences to cut and paste them together (Schatz and Ji, 2011). The 3D topology of the antigen receptor loci therefore must play a role in their regulation. 3D FISH studies were originally performed to search for topological features of the recombining loci. Indeed it was shown that the two ends of the receptor loci spatially come together prior to rearrangement (Kosak et al., 2002; Fuxa et al., 2004). The simultaneous visualization of intervening sequences then allowed demonstrating that locus contraction was not just a consequence of compaction but the result of chromatin looping, with intervening sequences looping out (Roldan et al., 2005; Sayegh et al., 2005; Jhunjhunwala et al., 2008). Multiple proteins including Pax5, YY1, CTCF, cohesin, and ikaros have been implicated in the spatial organization of these gene loci. Initial evidence for this was based on the observation that their depletion reduced contraction of the locus and lead to altered usage of the V genes during recombination (Roldan et al., 2005; Sayegh et al., 2005; Liu et al., 2007; Reynaud et al., 2008; Degner et al., 2009). More recently, 3C-based evidence was provided for looping between CTCF and cohesin bound chromatin sites across the antigen receptor loci (**Figure 1B**). Long-range chromatin interactions with three regulatory sequences in particular, the 3′ regulatory region (3′RR), the Eµ-intronic enhancer and the recently discovered intergenic control region 1 (IGCR1), seem important for proper rearrangement of the IgH locus. These loops may facilitate the inclusion of distal V genes, thereby enhancing the diversity of choice in usage of coding V elements during V(D)J recombination (Degner et al., 2011; Guo et al., 2011a, 2011b; Ribeiro de Almeida et al., 2011; Seitan et al., 2011). Additionally, CTCF and cohesin may regulate chromatin accessibility and transcription in sub-regions of the loci, thereby directing the recombination machinery. As was pointed out, while multiple proteins that shape the conformation of the antigen receptor loci are known now, there is as yet no evidence that they act directly to promote synapsis between distal gene segments (Seitan and Merkenschlager, 2012). Whether such activity exists, or whether the overall spatial structure of the antigen receptor loci is already sufficient to direct such interactions and warrant usage of the full repertoire of gene segments, remains to be investigated.

### H19/Igf2 LOCUS

The H19/Igf2 locus is an imprinted locus, with the H19 gene being expressed from the maternal and the Igf2 gene from the paternal allele. Both genes are under the control of a shared enhancer located on one side of the locus, 3′ of the H19 gene. The targeting of this enhancer to either one of the genes is determined by an imprinting control region (ICR) located in between Igf2 and H19 (Bartolomei et al., 1993; Ferguson-Smith et al., 1993; Leighton et al., 1995; Thorvaldsen et al., 1998). This ICR, which contains multiple CTCF binding sites, is methylated when paternally inherited and unmethylated when derived from the mother (Bartolomei et al., 1993; Ferguson-Smith et al., 1993). CTCF can only bind to the unmethylated, hence the maternally inherited, ICR (**Figure 1C**) (Bell and Felsenfeld, 2000; Hark et al., 2000).

Using an elegant approach that involved the site-specific integration of ectopic Gal-binding sites near the ICR it was shown that the ICR separates the H19 and the Igf2 gene in different chromatin compartments (Murrell et al., 2004). Because of the distinct capacity to bind CTCF, ICR contacts differ between the alleles such that enhancers are enabled to contact the Igf2 gene on the paternal allele but not on the maternal allele (Murrell et al., 2004). Subsequent studies based on 3C technology came to similar but not identical conclusions (Kurukuti et al., 2006; Yoon et al., 2007). Whereas one study reported bi-allelic interactions between the ICR and the enhancers (Kurukuti et al., 2006), another reported this interaction to be specific for the maternal allele. This study also showed that the CTCF-bound ICR promiscuously contacted enhancers and promoters, suggesting that such contacts are important for insulators to block effective enhancer–promoter communication (Yoon et al., 2007). In addition to its insulator function, the ICR appears required to initiate H19 gene expression: upon deletion of the four CTCF binding sites in the ICR, H19 transcripts were hardly detectable in the early embryo (Engel et al., 2006). In summary, studies on the H19/Igf2 locus confirm that gene competition for a shared enhancer involves competition for physical promoter–enhancer interactions. Moreover, they show that insulators bound by CTCF can hamper this interaction, possibly by physically competing for these contacts.

### 3D ORGANIZATION OF THE Hox GENES

When it comes to developmental gene regulation, the Hox gene clusters are among the most fascinating gene clusters. In mammals, four of these clusters are present (HoxA–D), each containing roughly a dozen genes that are expressed during development in a temporal and spatial manner that is co-linear with their genomic context (Kmita and Duboule, 2003). The HoxD gene cluster, but also other Hox clusters, is flanked on both sides by large gene-poor chromosomal regions. The Hox genes encode for transcription factors and are important for body axis formation as well as proper formation of the extremities. Correct spatiotemporal expression along the body axis appears controlled within the gene cluster proper, independent of surrounding gene sequences. As was shown by 4C technology, here the genes show little specific interactions with surrounding sequences, but fold into a distinct active and inactive compartment. When moving posteriorly along the axis, the number of genes contained within the active compartment increases, in agreement with their progressive activation and corresponding change of histone modifications (Noordermeer et al., 2011a). It was suggested that this topological separation can mediate the temporal expression pattern of the HoxD genes. In the extremities, in this case the developing limb bud, a different mechanism of transcriptional control is in place, with a correspondingly different 3D conformation of the gene cluster. The HoxD genes depend on distinct long-range regulatory sequences for their expression in the proximal and distal parts of the limb bud (**Figure 1D**). These sequences are present in the gene-poor regions located on the telomeric and centromeric side of the gene cluster, respectively (Spitz et al., 2003; Gonzalez et al., 2007). The active, much more than the inactive, HoxD genes loop toward these sides to contact the regulatory DNA sequences. Based on the DNA contact profiles of the active HoxD13 gene, as generated by 4C technology, new enhancers were identified in the gene desert that showed correct spatiotemporal reporter gene expression in transgenic mice (Montavon et al., 2011). The emerging picture from these studies is that Hox gene expression in the limb bud is under the control of a complex regulatory landscape with many enhancers spread over hundreds of kilobases of flanking DNA working in concert (Montavon et al., 2011). This picture seems confirmed by a recent high-resolution FISH study, which also revealed that further fine-tuning of the contacts between HoxD genes and flanking regulatory sequences takes place along the anterior–posterior axis of the limb bud (Williamson et al., 2012). A 5C analysis of the HoxA gene cluster in human primary fibroblasts taken from different anatomical sites revealed yet another dimension of Hox gene regulation. Contacts were identified with a site 5′ of the cluster that expresses a long intergenic non-coding RNA (lincRNA), named HOTTIP (Wang et al., 2011). HOTTIP RNA was reported to recruit proteins (WDR5) necessary to modify the histones and activate transcription of the genes contacted by the lincRNA locus (Wang et al., 2011). Thus, proper spatiotemporal Hox gene expression appears to be controlled by a very complex network of proximal and distal regulatory sequences that loop in a developmentally controlled manner toward specific Hox genes to physically confront them with activating protein and RNA molecules.

## THE OVERALL SHAPE OF THE 3D GENOME

The initial 3C studies discussed above focused on individual genes and gene clusters, highlighting the functional importance of local chromatin loops and uncovering proteins that determine the topology of these gene loci (Splinter and de Laat, 2011). However, the genome is structurally organized also beyond the level of individual gene clusters. Original evidence that overall chromatin in the nucleus is not organized in a random fashion and that nuclear organization is related to transcriptional activity comes from microscopy observations. It showed the separation of densely packed inactive chromatin and loosely packed active chromatin and demonstrated that chromosomes occupy individual chromosome territories (CTs; Branco and Pombo, 2006; Joffe et al., 2010). It also demonstrated that larger chromosomes tend to occupy more peripheral positions in the nucleus, while smaller ones often reside more in the nuclear interior. A recurrent theme in nuclear organization is that folding and positioning follow probabilistic rules. Thus, a given chromosome will have a preferred nuclear position, but this does not imply that it occupies this exact position in every cell (Bolzer et al., 2005). In other words: all genomes in a population of cells can be expected to fold according to the same probabilistic rules, yet every single cell likely has a different genome structure. Thanks to the development of more genome-wide versions of 3C technology (de Wit and de Laat, 2012; Dostie and Bickmore, 2012), the underlying, probabilistic, rules for genome folding are now rapidly being uncovered.

**FIGURE 2:**
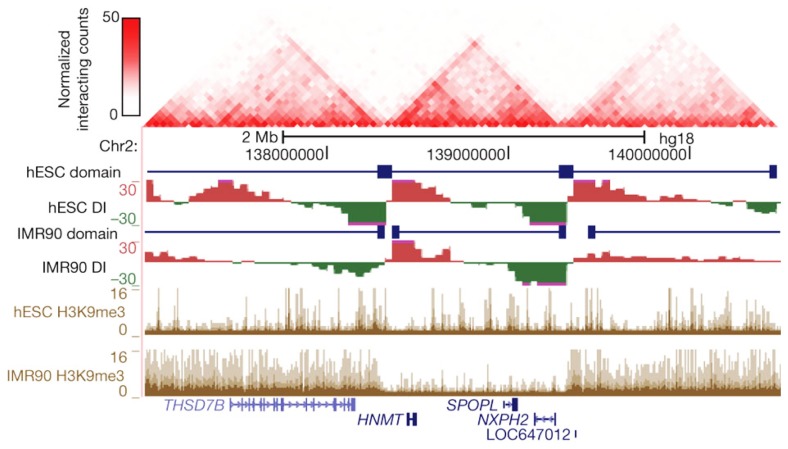
**Topological boundaries can act as barriers for spreading of heterochromatin.** The 2D heat map shows the Hi-C interaction frequency in human ES cells. Underneath is indicated the directionality index (DI) in hESCs and IMR90 cells. The DI is a Hi-C measure showing a site’s preference to engage in unidirectional contacts with downstream (red) or upstream (green) sequences. Borders of the topological domains are defined by a change in the directionality of interactions (transition from green to red). The UCSC Genome Browser shots show the distribution of H3K9me3, a measure for heterochromatin formation. Note that in IMR90 cells heterochromatin stops at the topological boundaries. Reprinted by permission from Macmillan Publishers Ltd (Dixon et al., 2012), copyright (2012).

The most dominant force shaping the 3D genome seems the spatial separation between active and inactive chromatin. First observed under the microscope as a general feature of nuclear organization, it was then confirmed to also be relevant for the folding of individual chromosome segments (Shopland et al., 2006) and, at much higher resolution, for the genomic environments of individual genes (Simonis et al., 2006). The latter observation made by 4C technology for a few selected chromosomal sites was confirmed to apply to regions across the genome by recent Hi-C studies. In Hi-C, all versus all interactions of the genome are mapped, with the resolution of contact maps depending on the depth of sequencing, the size of the genome, and the complexity of the sample analyzed (Lieberman-Aiden et al., 2009; Yaffe and Tanay, 2011; Dixon et al., 2012; Kalhor et al., 2012). Hi-C studies showed that chromosomes are subdivided into topological domains that cover 0.2–1 Mb. The domains mark chromosomal regions within which DNA contacts are confined. They generally demarcate regions with a defined gene density and activity, and with corresponding chromatin accessibility, histone modifications, and replication timing. Preferred contacts among two types of topological domains are seen, the active and inactive topological domains, with the separation of active and inactive chromatin in the nucleus as a consequence (Lieberman-Aiden et al., 2009; Yaffe and Tanay, 2011; Dixon et al., 2012; Kalhor et al., 2012; Nora et al., 2012). In Drosophila in particular, an additional domain type hallmarked by the association of polycomb group (PcG) proteins is observed, which also shows preferred contacts with other PcG-bound topological domains (Tolhuis et al., 2011; Sexton et al., 2012). Marks for active chromatin (DNase I sensitivity, H3K4me1 and -me3, RNAPII) were enriched for regions showing also interchromosomal DNA contacts (Yaffe and Tanay, 2011; Kalhor et al., 2012), suggesting that open and active chromatin most easily reaches out of the CT. Boundaries of the domains were found enriched for CTCF, H3K4me1, transcriptional start sites (TSSs) and housekeeping genes, tRNA genes and SINE elements (Yaffe and Tanay, 2011; Dixon et al., 2012; Sexton et al., 2012). Interestingly, during cellular differentiation the topological domains appear to largely remain intact and structural changes mostly occur within the domains, suggesting that the domain boundaries are largely conserved between cell types (Dixon et al., 2012; **Figure 2**). The active and inactive compartments each seem to organize themselves independently. This was shown in studies on the active and inactive X chromosome in mammalian female cells, where the inactive X chromosome showed normal contacts between active chromatin regions but was found to specifically lack long-range contacts between inactive chromatin domains. Interestingly, these latter contacts were restored when the non-coding RNA Xist, which coats the inactive X chromosome, was deleted, implicating a role also for non-coding RNA in chromosome topology (Splinter et al., 2011).

Whether RNA plays a general role in the topological organization of chromosomes remains to be demonstrated. Proteins, however, are known to shape the configuration of the genome inside the cell. Nuclear lamina proteins, CTCF, cohesin, and RNAPII are best recognized as general organizers of the 3D genome and will be discussed below.

## PROTEINS SHAPING THE GENOME

### LAMINS AND THE NUCLEAR PERIPHERY

The nuclear periphery of mammalian cells is known to be enriched for inactive chromatin and to correlate with relatively low gene expression levels (Brown et al., 1997, 1999; Skok et al., 2001; Zink et al., 2004). The inner part of the nuclear membrane is coated with a protein network called the nuclear lamina. Lamina-associated domains (LADs), spanning 0.1–10 Mb, were identified across the genome based on an elegant approach called DamID, which takes advantage of DNA adenine methylase (DAM) fused in this case to lamin B1, a component of the nuclear lamina (Guelen et al., 2008). Characterization of the genomic content enriched in LADs showed that they are generally gene poor, transcriptionally inactive, depleted for active transcription marks such as RNAPII and active histone marks. At LAD borders, promoters transcribing away from LADs are found enriched, as well as CTCF binding sites (Guelen et al., 2008). Dynamic interaction of the genome with the nuclear lamina was seen during neural differentiation of embryonic stem cells (ESCs). Some, but certainly not all, regions in the genome that were transcriptionally activated or repressed during this process changed their association to the nuclear lamina accordingly (Peric-Hupkes et al., 2010). Furthermore, mis-expressed genes were correlated with a change in nuclear localization of these genes in cells carrying disease related lamin A mutations (Mewborn et al., 2010). Recently, mapping of the lamin A-interacting genes showed that lamin A is similarly, involved in anchoring silent genes to the nuclear lamina. Intriguingly though, depletion of lamin A changed the nuclear positioning of the lamin A bound genes but was not enough to change the expression of these genes (Kubben et al., 2012). Oppositely, as discussed below, the artificial tethering of genes to the nuclear lamina sometimes, but not always, leads to their silencing. Clearly, the nuclear lamina is involved in the spatial organization of the genome in a manner that at least reflects transcriptional activity. To what extent a peripheral positioning also determines gene activity still remains to be investigated.

### CTCF

CTCF is probably the best characterized structural organizer of the genome to date. From the first description of the protein (Lobanenkov et al., 1990), it has been shown to be a versatile protein having direct transcriptional effects (Filippova et al., 1996; Vostrov and Quitschke, 1997; Yang et al., 1999) as well as effects on transcription over distance (Bell et al., 1999). The approximately 40,000 CTCF binding sites in the human and murine genome preferentially locate to intergenic regions and show high conservation between different cell types (Barski et al., 2007; Kim et al., 2007; Chen et al., 2008; Hou et al., 2010). CTCF is ubiquitously expressed and an essential protein (Heath et al., 2008). It has a well established role in chromatin folding at the β-globin locus, and in chromatin folding and gene expression at the H19/Igf2 locus and the antigen receptor loci, as described above. Also at other loci, including the human major histocompatibility complex (MHC) class II locus and the Kcnq5 gene, CTCF-mediated chromatin loops were found involved in gene regulation (Majumder et al., 2008; Majumder and Boss, 2010; Ren et al., 2012). At a more genome-wide scale, CTCF binding sites were found enriched at borders between the topological domains identified by Hi-C (Yaffe and Tanay, 2011; Dixon et al., 2012) as well as at LAD borders (Guelen et al., 2008), further hinting at an important role for this protein in organizing the 3D structure of chromosomes. Interest in the protein was raised even further when cohesin was found to co-occupy genomic sites with, and be positioned by, CTCF (see below; Parelho et al., 2008; Rubio et al., 2008; Wendt et al., 2008).

**FIGURE 3:**
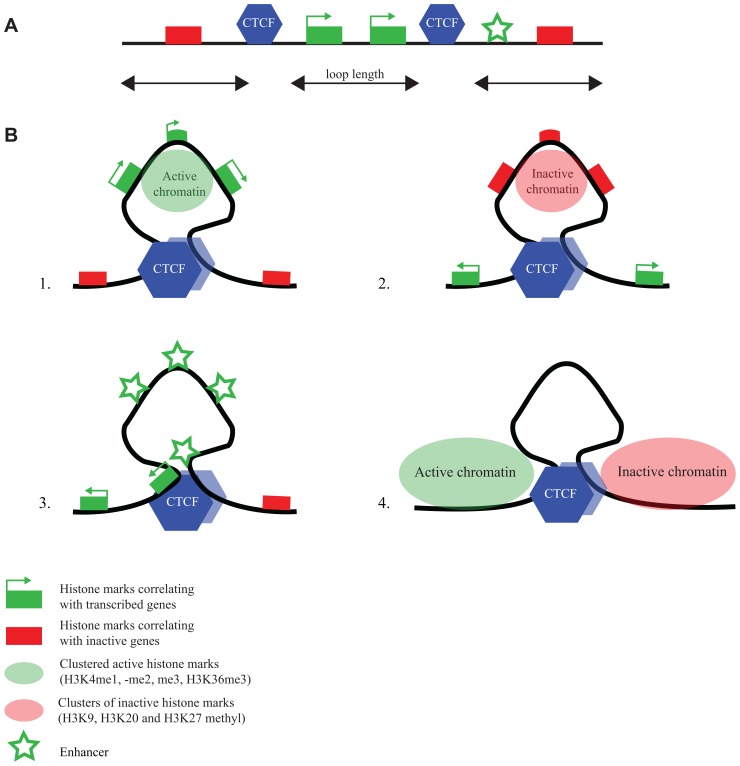
** CTCF flanks chromatin marked by specific histone modifications.** **(A)** Linear representation of a chromosomal region with active and inactive genes, CTCF binding sites and an enhancer (for explanation of symbols, see bottom figure). **(B)** ChIA-PET reveals different chromatin loops formed by CTCF (Handoko et al., 2011): CTCF loops demarcate regions **(1)** with active chromatin marks, **(2)** with inactive chromatin marks, **(3)** with enhancers and promoters, and **(4)** with undefined chromatin surrounded by regions with opposing chromatin signatures.

ChIA-PET is a technology that combines chromatin immunoprecipitation (ChIP) with a 3C approach, to direct DNA topology studies specifically to the genomic sites that are bound by a protein of interest (Fullwood et al., 2009). ChIA-PET was applied to CTCF to study its DNA interactome (Handoko et al., 2011). Mostly intrachromosomal and a few interchromosomal interactions between CTCF-bound sequences were identified, with the intrachromosomal loop sizes ranging from 10–200 kb. The loops appeared to serve different purposes (**Figure 3**). They can isolate an active chromatin region from surrounding inactive chromatin or bring together enhancers and promoters in a single loop. Yet other loops formed by CTCF seem to isolate undefined chromatin from a flanking active and inactive chromosomal region (Handoko et al., 2011). Only a few percent of the total number of CTCF sites was found engaged in loop formation. This suggests that ChIA-PET only uncovers the tip of the topological iceberg. Alternatively, the majority of CTCF-bound sites is not involved in long-range chromatin interactions. If the latter is true, it would be interesting to understand what determines whether a CTCF binding site is engaged or not in a chromatin loop.

### COHESIN

Cohesin is a multiprotein complex that forms a ring-like structure which captures and holds together the two DNA double-strand helices of sister chromatids after DNA replication. The discovery that cohesin binds to CTCF binding sites also in G1 phase of the cell cycle suggested that it has an additional role besides keeping sister chromatids together. Without CTCF, cohesin still binds to chromatin but is no longer found at specific locations along the chromosome arms, suggesting that CTCF positions cohesin on the chromatin (Parelho et al., 2008; Rubio et al., 2008; Wendt et al., 2008). Given its shape and function, cohesin was obviously considered an attractive protein for chromatin loop formation (Nasmyth and Haering, 2009). Indeed, cohesin was found to mediate chromatin looping at CTCF binding sites in several loci including the immunoglobulin locus (Degner et al., 2009), the interferon gamma locus (Hadjur et al., 2009), the HoxA locus (Kim et al., 2011), the MHC class II locus (Majumder and Boss, 2011), the β-globin locus (Hou et al., 2010; Chien et al., 2011), and the H19/Igf2 locus (Nativio et al., 2009). Interestingly, at several sites bound by CTCF across different cell types, cohesin association was found to differ in a cell-dependent manner, with topological changes and altered gene expression changing accordingly (Chien et al., 2011; Kim et al., 2011). This suggests that possibly the co-recruitment of additional factors like cohesin determines whether a given CTCF binding site is engaged in a chromatin loop in a given cell type. A CTCF-independent role for cohesin in transcription regulation was also demonstrated, in a study that revealed cohesin and estrogen receptor co-binding near upregulated genes upon estrogen treatment of MCF-7 cells (Schmidt et al., 2010). Cohesin binding was enriched at sites demonstrated by ChIA-PET to form ER-mediated loops (Fullwood et al., 2009), suggesting that cohesin may help ER to mediate transcriptional responses via long-range DNA interactions (Schmidt et al., 2010). A further CTCF-independent role of cohesin was observed in ESCs, where cohesin association was detected at sites bound by mediator and RNAPII, but not CTCF (Kagey et al., 2010). Enhancer promoter interactions of tissue-specific genes were shown by 3C technology to be mediated by the interaction with mediator and the cohesin loading factor, Nipbl. Cohesin and mediator together share distinct genomic sites in different tissues, unlike the shared binding sites between CTCF and cohesin which seem largely conserved between cell types (Kagey et al., 2010). Thus, cohesin may have CTCF-dependent and -independent roles in chromosome topology and gene regulation during development (Kagey et al., 2010; Schmidt et al., 2010).

### RNA pol II

Transcription, and in particular the nuclear localization of RNA polymerase, has always been considered an attractive candidate to shape the 3D genome (Fraser and Bickmore, 2007). It may explain why active chromatin comes together in the nuclear space. Clusters of RNAPII, termed transcription factories, have been identified in the nucleus by electron microscopy and immunofluorescence (Jackson et al., 1993; Iborra et al., 1996; Grande et al., 1997; Jackson et al., 1998). It is difficult to assess the number of factories per cell as this appears to differ between cell types and is also dependent on the microscopy method used (Osborne et al., 2004). The concept assumes that genes need to migrate to pre-existing protein factories where multiple genes are transcribed simultaneously. In a more extreme model there may even be dedicated transcription factories that contain specific combinations of transcription factors and therefore need to be visited by defined categories of co-regulated genes (Xu and Cook, 2008; Schoenfelder et al., 2010). Does form indeed follow function, as suggested by these models? Not all observations necessarily support this idea. Live cell imaging with fluorescently tagged RNAPII so far has not provided convincing evidence for the existence of transcription factories (Kimura et al., 2002; Zobeck et al., 2010), nor for movement of genes upon transcriptional activation (Zobeck et al., 2010). Inhibition of transcription caused most RNA polymerase to dissociate from active genes, yet had no appreciable impact on their contacts with other active genes, as assessed by 4C technology, nor interfered with enhancer–gene contacts (Palstra et al., 2008). The recent demonstration that loop formation in the β-globin locus precedes transcriptional activation also suggests that function follows form (Deng et al., 2012). Possibly, shape and function both influence each other. It was proposed that initiating RNA polymerases that are close together in the nuclear space may aggregate to form the observed transcription factories. This is easiest envisioned to happen between genes that are proximal on the linear chromosome, as these per definition are close together in the nuclear space, rather than involving genes searching for distant co-regulated genes (Razin et al., 2011). Indeed, a ChIA-PET study focusing on chromatin loops formed between RNAPII-bound chromatin sites recently demonstrated the clustering of active gene promoters that neighbor each other on the chromosomes (Li et al., 2012).

ChIA-PET enables an unbiased genome-wide assessment of contacts formed by the genomic sites bound by a protein of interest. Remarkably, for all proteins studied so far, ChIA-PET primarily identifies local contacts between sites close together on the linear chromosome. On the one hand this probably emphasizes the importance of local chromatin loops for the expression of genes involved in these loops. On the other hand it raises the question: how important is the position of a gene relative to other chromosomal regions elsewhere in the genome? So far, mostly microscopy studies have tried to address this.

## GENE POSITIONING IN THE CELL NUCLEUS

One of the earliest studies that followed the positioning of individual genes focused on the Ikaros proteins, required for the development of cells of the lymphoid lineage (Brown et al., 1997, 1999). Highly expressed lymphoid genes like CD45 and CD19 were not found associated with Ikaros in B cells, but stage-specific genes showed differential association with Ikaros during differentiation (Brown et al., 1997). When bound by Ikaros, these genes were found to be silenced and repositioned to pericentromeric heterochromatin (PCH). It was proposed that PCH-association facilitated heritable gene silencing during B cell differentiation (Brown et al., 1997, 1999). Subsequently, also other genes were found to occupy particular nuclear locations in relation to their status of transcription, and again this has been studied most notably for the forementioned model gene loci. The IgH locus, for example, was found to adopt a peripheral position in cells not transcribing the gene. When active in B cells, it adopts a more internal nuclear position (Kosak et al., 2002). In mature B cells, the non-productive IgH allele was reported to be frequently associated with PCH, perhaps to ensure its silencing (Skok et al., 2001; Roldan et al., 2005). Repositioning of loci to PCH is also important during lineage choice in T cells (Merkenschlager et al., 2004; Collins et al., 2011), where repositioning of the CD8 locus to PCH is seen in CD4^+^ T cells and vice versa. Here localization was stated to be predictive for the developmental state of the T cell (Merkenschlager et al., 2004). Localization of inactive genes to the nuclear periphery was also found for the human CFTR locus (Zink et al., 2004; Ballester et al., 2008) and the casein cluster in mammary glands (Kress et al., 2011).

Similar observations were done on the β-globin locus. During erythroid maturation, which is accompanied by LCR-mediated transcriptional activation, the locus was observed to move from the periphery to the interior. Expression at the periphery was found, but it occurred more frequently in the nuclear interior, and the inward movement was dependent on the LCR (Ragoczy et al., 2006). Whereas one study reported preferred clustering of the active β-globin genes with other active erythroid genes (Schoenfelder et al., 2010), two other studies did not find this (Simonis et al., 2006; Brown et al., 2008). A different type of movement was observed for the Hox gene clusters. Induction of Hox gene expression influenced the position of the Hoxb1 and Hoxb9 genes relative to their CTs (Chambeyron et al., 2005). Expression was associated with a position more outside of the CT. This nuclear organization was dynamic as hoxb1 and -b9 could be repositioned in different stages of differentiation, in agreement with their transcriptional state (Chambeyron and Bickmore, 2004; Chambeyron et al., 2005). Similarly, Hoxd genes were looped outside their CT in the tailbud of e9.5 mice (Morey et al., 2007). In the forelimb bud, where Hoxd9 is also expressed (Tarchini and Duboule, 2006), no looping out of the CT for this gene is found (Morey et al., 2007). Moreover, neighboring genes that are dragged along outside the CT not necessarily show bystander upregulation of gene expression (Noordermeer et al., 2008; Morey et al., 2009). Thus, these studies show that genes can, but do not need to move away from their CT and that looping out of the CT is not sufficient for gene activation.

To better understand the consequences of nuclear repositioning, tethering experiments can be done. These are based on the genomic integration of repeats of DNA binding sites (often bacterial LacO or TetO sequences) and the simultaneous expression in eukaryotic cells of cognate bacterial proteins (LacR or TetR) fused to a protein of interest. Fusion to fluorescent GFP enables following the genomic integration sites in live cell imaging studies (Robinett et al., 1996; Tumbar et al., 1999) and revealed that individual gene loci show limited movement during the interphase of mammalian cells (Chubb et al., 2002). Recruitment of transcriptional activators caused locus decondensation concomitant with increased transcription and histone acetylation, but neither was required to maintain the decondensed chromatin state (Tumbar et al., 1999; Ye et al., 2001; Nye et al., 2002; Chen et al., 2004). The targeting of heterochromatin protein 1 (HP1) to a non-heterochromatic locus reduced gene expression, induced locus condensation, and resulted in local H3K9me3 modifications, indicative of heterochromatin formation (Verschure et al., 2005; Hathaway et al., 2012).

Several studies used fusions of lamina components to address the consequences of recruitment to the nuclear periphery. In one study, which also enabled simultaneous visualization of nascent transcripts, the association of lamin B1 to a reporter locus caused repositioning, but only after cell division. Here, the kinetics of gene activation were similar to that at internal locations, indicating that loci maintain their transcriptional competence at the nuclear periphery (Kumaran and Spector, 2008). In another study, however, repositioning through the recruitment of emerin (EMD) was found to be accompanied by reporter gene silencing (Reddy et al., 2008). A third study measured chromosome-wide gene expression differences after tethering of the chromosome to the inner nuclear membrane. A few genes, some nearby and some at great distance from the integrated LacO cassettes, showed repressed transcription, but expression was not incompatible with peripheral location (Finlan et al., 2008). Interestingly, in a recent study it was demonstrated that the ectopic integration of LAD sequences can also reposition surrounding chromosomal regions to the periphery, and negatively influences the expression of surrounding genes (Zullo et al., 2012). GAGA motifs were found enriched in LADs and demonstrated to be responsible for peripheral recruitment. They are targets for the transcriptional repressor cKrox and the associated HDAC3 and Lap2β proteins, which were found to be necessary for peripheral recruitment (Zullo et al., 2012). Collectively, these studies suggest that nuclear compartmentalization and gene expression are coupled, but also emphasize the probabilistic nature of nuclear organization: genes positioned at the periphery of the cell nucleus do not necessarily lose their capacity to be transcribed, but appear more susceptible to transcriptional repression than at more internal nuclear positions.

## CONCLUDING REMARKS

Over the last years research has made major progress in understanding the relationship between structure and function of the genome. Studies on model gene systems such as those discussed here have shown that local DNA interactions between regulatory sites and genes are important for transcriptional control. In mammals, such regulatory interactions can take place over chromosomal distances as large as a megabase. Transcription factors bound to these chromatin sites seem responsible for setting up the chromatin loops in chromosomal segments. Others, such as CTCF, appear capable to modify chromatin topology such that it hampers these interactions. Beyond this local scale of structural organization, genome folding seems to follow more probabilistic rules. Active and inactive chromatin separate, some chromosomal regions have an increased chance of being at the periphery than others, and, when assayed across large cell populations, all individual gene loci appear to have many different contact partners. Together this suggests that the exact genome conformation will differ from cell to cell. As a consequence, a given contact between two dispersed genomic regions will only occur in a subset of cells. If this contact influences the expression of the associated genes, this may not have an impact on the entire cell population, but can be important for the individual cells involved, as was shown recently (Noordermeer et al., 2011b). To study the functional consequences of cell to cell differences in genome conformation we therefore probably need to analyse form and function at the single cell level, with the exciting possibility to discover that the overall shape of our genome can determine cell fate decisions of individual cells.

## Conflict of Interest Statement

The authors declare that the research was conducted in the absence of any commercial or financial relationships that could be construed as a potential conflict of interest.
